# Nurse‐Led Telephonic Care Following Emergency Department Visits for Persons Living With Dementia and Their Care Partners: A Program Description

**DOI:** 10.1111/jgs.70429

**Published:** 2026-05-21

**Authors:** Valerie T. Cotter, Corita R. Grudzen, Janessa Griffith, Allison Cuthel, Afshana Hoque, Alicia I. Arbaje, Cameron J. Gettel, Aditi Durga, Ariana Emami, Keith Goldfeld, Joshua Chodosh, Manish N. Shah, Abraham A. Brody, ED‐LEAD Investigators, Lauren Abbate, Rebecca Anthopolos, Fernanda Bellolio, Jonathan Berkowitz, Andrea Blome, Erik Jonathan Blutinger, Justin Kenneth Brooten, Reed Caldwell, Christopher Caspers, Nathaniel Chin, Edward Cisek, Jeremy Thomas Cushman, Dan David, Allison L. Ducharme‐Smith, Scott Dresden, Mary Ellen Dupont, Marie Carmelle Elie, Andra Farcas, Bucky Ferozan, Jori Fleisher, Nicholas Genes, Andrea Gilmore‐Bykovski, Scott Goldberg, Elizabeth Goldberg, Satheesh Gunaga, Heather Heaton, Courtney Jones, Maura Kennedy, Timmy Li, Joseph Miller, Brian Mittman, Kei Ouchi, Jennifer Portz, Alex Quinones, Suzanne Ryer, Ashley Shreves, Michelle Simpson, Silas Smith, Payal Sud, Joe Suyama, Scotty Thomas, Victoria Vaughan Dickson, Brian W. Patterson, Ian Wittman, Nancy Wood

**Affiliations:** ^1^ Johns Hopkins, School of Nursing Baltimore Maryland USA; ^2^ Department of Psychiatry and Behavioral Science Johns Hopkins School of Medicine Baltimore Maryland USA; ^3^ Department of Supportive and Acute Care Services Memorial Sloan Kettering Cancer Center New York New York USA; ^4^ Division of Geriatric Medicine and Gerontology, Department of Medicine Johns Hopkins School of Medicine Baltimore Maryland USA; ^5^ Department of Emergency Medicine Yale School of Medicine New Haven Connecticut USA; ^6^ New York University Rory Meyers College of Nursing New York New York USA; ^7^ Department of Population Health New York University Grossman School of Medicine New York New York USA; ^8^ Division of Geriatric Medicine and Palliative Care New York University Grossman School of Medicine New York New York USA; ^9^ VA New York Harbor Healthcare System New York City New York USA; ^10^ BerbeeWalsh Department of Emergency Medicine University of Wisconsin‐Madison Madison Wisconsin USA

**Keywords:** advance care planning, behavioral and psychological symptoms of dementia, dementia care, pragmatic clinical trial, telephonic care

## Abstract

Most visits to the emergency department (ED) by persons living with dementia (PLWD) who are then discharged back into the community are preventable. However, care partners and other caregivers of the over 6 million PLWD residing in the United States lack the supports, services and timely access to clinical care to address many common needs in the community. Thus, care partners resort to taking the PLWD to the ED, a sub‐optimal environment that can be traumatic to the PLWD, increase iatrogenesis, and ultimately may not resolve the underlying reason for the visit. Longitudinal nurse‐led telephonic care (NLTC) provided following an ED visit with community discharge may present an effective and efficient model for health systems to support care partners, decant busy EDs, and provide high‐quality, person and family‐centered impactful care to support PLWD and their care partners. This paper describes the development of an NLTC program being implemented as part of the Emergency Departments LEading Transformation of Alzheimer's and Dementia Care (ED‐LEAD) trial, a factorially designed embedded pragmatic clinical trial in 79 EDs. The NLTC program utilizes components of two previously tested programs, the Aliviado Dementia Care quality improvement program, and the Emergency Medicine Palliative Care Access (EMPallA) nurse‐led telephonic palliative and transitional care program to support PLWD and their care partners. Successful implementation of the NLTC program may lead to increased uptake of NLTC programs by health systems, improving quality of care and quality of life for PLWD and their care partners.

## Summary

- Key points
  ○ Most visits to the emergency department (ED) by persons living with dementia (PLWD) who are then discharged back into the community are preventable.
  ○ While dementia case management programs have been implemented as part of efficacy trials, few have tested real‐world implementation and uptake as part of an embedded pragmatic clinical trial.
  ○ Nurse‐led telephonic care is a scalable, potentially cost‐effective method for population health management of PLWD and care partner support.
- Why does this paper matter?
  ○ By initiating nurse‐led telephonic care after an initial ED visit, health systems can identify PLWD who are at high risk for future utilization and their care partners and provide targeted supports earlier to address patient, care partner, and health system needs.

## 1 Background and Significance

More than 50% of older adults with Alzheimer's disease and Alzheimer's disease‐related dementias (AD/ADRDs) visit the emergency department (ED) each year [1, 2]. These individuals are seriously ill, have significant healthcare costs (e.g., hospital admissions, repeat ED visits), and face increased mortality [1, 3]. Most persons living with dementia (PLWD) live in the community, and those with serious illness experience frequent transitions and complex interactions with the healthcare system. Care partners of PLWD experience poor communication, and lack of engagement during the ED discharge process; challenges during the acute illness and recovery phases; and difficulty navigating the health care system after an ED encounter [4]. Interventions that are pragmatic, feasible, and scalable are urgently needed to assist seriously ill PLWD and their care partners through advance care planning, behavioral and psychological symptoms of dementia (BPSD) management, transitional care, and case management [5]. This paper describes one of three interventions being tested in Emergency Departments LEading Transformation of Alzheimer's and Dementia Care (ED‐LEAD), a cluster‐randomized multifactorial pragmatic trial focused on any individual identified with dementia who visits and is discharged directly from the ED (see related editorial in this issue by Corita R. Grudzen and analytic plan published elsewhere) [6, 7]. The study seeks to test alone, or in combination three discrete but potentially complementary interventions, ED Care Redesign, Community Paramedicine Transition Intervention, and Nurse‐Led Telephonic Care (NLTC). This purpose of this paper is to describe the NLTC intervention, which provides post‐discharge support to PLWD and their care partners following an ED visit.

## 2 Background Evidence for the Intervention

When asked about their priorities, seriously ill PLWD and their care partners express that they would prioritize the following: (1) planning for the PLWD's future; (2) maximizing the PLWD's cognitive and physical abilities to allow them the greatest independence and comfort; and (3) care partner support [8, 9]. Yet, seriously ill PLWD have higher BPSD rates [10] and low rates of documented advance care planning [11] compared with those earlier in the disease trajectory, and their care partners have twice the risk of high strain [12]. Telephonic programs, largely in earlier stages of AD/ADRD, have shown efficacy in addressing these priorities. The most prominent of these are Care Ecosystem, a telephonic program focused on advance care planning, BPSD, and caregiver needs [13], and the UCLA Comprehensive Disease Management Program [14], an initial in‐person comprehensive assessment with telephonic follow‐up by community‐based organizations to monitor implementation and changing needs [15].

Telephonic care increases access to high‐quality care and has the potential to reduce substantial inequities that exist in care for PLWD and their care partners. Global racial and ethnic inequities and social determinants of health play a large role in perpetuating inequities in PLWD and their care partners [16]. Telephonic interventions can screen and address social determinants of health to improve outcomes [17, 18], including through nurse‐led care, thus reducing ED visits [19, 20].

## 3 Aim

This paper aims to describe the conceptual basis for a NLTC intervention and the details of its design, including the training and setting involved in its delivery, as well as the process measures monitored to ensure delivery.

## 4 Intervention Design

### 4.1 Overview of the Intervention

This study combines two previously tested interventions, Aliviado Dementia Care and the Emergency Medicine Palliative Care Access (EMPallA) nurse‐led telephonic palliative and transitional care program into a combined Aliviado Dementia Care‐Telephonic Edition intervention [21, 22, 23, 24, 25, 26, 27, 28, 29]. These programs were merged as the Aliviado program includes dementia care specific competencies but had only previously been delivered through home‐based care, and the EMPallA program which focused on telephonic nurse delivered palliative care and case management but excluded care for PLWD. The combined program was successfully tested with a group of telephonic nurses for acceptability, usability, and feasibility across two sites.

### 4.2 Aliviado Dementia Care

Aliviado Dementia Care, initially developed for home health and hospice agencies, is a quality improvement program that focuses on an evidence‐based practice approach to caring for PLWD and care partners in the community. The program is interdisciplinary, helping clinical staff to develop an understanding of AD/ADRD in serious illness, learn to assess for transitional safety issues, assess and manage pain and BPSD, and communicate and problem‐solve effectively with the PLWD and care partners. It was conceptually guided by the Structural Model for Caregiving Stress [30]. Aliviado Dementia Care includes a mixture of live and asynchronous training that is discipline‐specific; a toolbox of assessment instruments, care plans, and care partner education materials written in English and Spanish at a sixth to eighth grade reading level; and a symptom treatment algorithm. The clinical team implements standardized care plans that support the implementation of evidence‐based strategies for: care transitions, safety issues, acute delirium, care partner strain, and BPSD management as part of their routine care visits. A resource manual tailored to the individual community is provided to address patient social needs and social determinants of health. A digital platform sends care partner education materials electronically, performs assessments, implements care plans, and reminds clinicians through mobile push notifications and emailed behavioral economic nudges and mentoring to maintain fidelity.

### 4.3 Preliminary Work

Before our current full‐scale embedded pragmatic clinical trials in home health and hospice, we performed a case‐controlled trial at two divisions of a large home healthcare agency (HHA) and pragmatically collected data for PLWD on admission and at the first 60‐day recertification or discharge from home healthcare in 2013. Throughout the study period, 158 PLWD were seen by the control site and 174 by the intervention site. Subjects were older, primarily spoke English or Spanish, and were Medicare or dual Medicare/Medicaid insured. The only significant baseline difference between the groups was the presence of fewer Black/African American participants in the control group (51.7% vs. 34.2%; *p* < 0.001). In the intervention group, we found increases in pain recognition at baseline (adjusted odds ratio [AOR] = 2.58; *p* < 0.001), pain reduction at 60 days (AOR = 1.99; *p* < 0.01), and identification of behavioral symptoms (AOR = 1.67; *p* < 0.05). We also found significantly increased analgesics use (primarily nonopioid analgesics) (AOR = 2.01; *p* < 0.05) and substantially decreased antipsychotic use (AOR = 0.53; *p* < 0.05) [31]. Additionally, there were fewer hospitalizations in the intervention cohort (12.2% vs. 21.2%, *p* = 0.05), although we were underpowered for multivariate analysis and could not control for clustering effects. In early data from our hospice trial, we found that individual hospices were able to reduce antipsychotic use, increase use of respite care to prevent care partner burnout, and reduce burdensome transitions at the end of life including hospitalizations.

### 4.4 EMPallA Nurse‐Led Telephonic Palliative and Transitional Care Program

The EMPallA nurse‐led telephonic palliative and transitional care program is an ED‐initiated 6‐month program that focuses on engaging persons with serious illness (excluding AD/ADRD) and their care partners in advance care planning, symptom management, case management, and facilitation with their provider team. The program was derived from the Senior Care Action Network (SCAN) Health Plan and modified to create more structure for the nurses in implementing the program. The nurse performs an initial structured telephonic assessment. After the initial call, the nurse presents the case at an interdisciplinary team meeting involving a palliative physician, geriatric and palliative nurse practitioner, other telephonic team nurses, and, where needed, a social worker and/or chaplain. The nurse develops three patient and care partner–centered problem–intervention goal care plans that are reviewed and updated during the interdisciplinary team meeting. These goals are addressed in follow‐up calls that occur at variable frequencies based on the needs of the PLWD and care partner; inbound calls from the PLWD and care partner can be included as needs arise. The nurse can bring the case back to other team members either during follow‐up team meetings or 1:1 when challenges arise, where they can benefit from additional input into the care plan.

### 4.5 Preliminary Work

A recent study compared the effectiveness of nurse‐led telephonic palliative care versus specialty outpatient palliative care on quality of life, symptom burden, loneliness, and healthcare use, after attending the ED [32]. Subjects were highly diverse (32% Black/African American, 11% Hispanic). About 19% of patients were lost to follow‐up during the first 6 months, 15% died, and 65% completed the 6‐month follow‐up. This includes hard‐to‐reach undomiciled individuals and others coming from disadvantaged backgrounds. More patients in the telephonic arm engaged in care (68%) than the outpatient arm (53%), suggesting that telephonic care may be more accessible to patients. Both arms showed small improvements in quality of life and decreases in symptom burden over time, despite disease progression; however, there were no clinically meaningful differences in the change in quality of life between the treatment arms.

## 5 Telephonic Intervention Description

### 5.1 Overview of the Combined Intervention

Aliviado Dementia Care‐Telephonic Edition is a protocolized intervention that combines training, care plans, assessment instruments, a BPSD treatment algorithm, and care partner education materials from the original Aliviado Dementia Care program, delivered through a structured initial assessment and follow‐up phone calls, care planning process, motivational interviewing training, and weekly interdisciplinary team meetings that follow original EMPallA program processes. Also added was a learning network of the nurses implementing the program across the country for nationwide program support and cohort and community building.

### 5.2 Training

All training was initially created through participatory research with an interprofessional team of clinicians, then refined through feedback from clinicians who completed the training. Unlike many AD/ADRD programs, Aliviado Dementia Care was culturally tailored for use in diverse settings and tested within multiple racial and ethnic minority populations across the United States, including multiple Hispanic, Black/African American, Afro‐Caribbean, and Asian sub‐groups. The evidence‐based dementia‐specific training includes four 1‐h modules, as well as 2‐h of motivational interviewing; the NLTC orientation and NLTC kickoff meeting are intended for implementation and operational readiness (Table 1).

**TABLE 1:** Aliviado dementia care‐telephonic edition training modules.

Training component	Objective	Time
NLTC orientation	Introduction to NLTC and training expectations	1 h
Aliviado training	Modules/Learning Objectives	4 h
	**Module 1: Overview of Dementia** Describe the significance and consequences of Alzheimer's Disease and Related Dementias Understand key differences in different types of Alzheimer's Disease and Related Dementias Describe an overarching approach to supporting persons living with dementia their care partners, and other caregivers	
	**Module 2: Assessing and Managing Pain in Persons Living with Dementia** Pain Assessment in Persons Living with Dementia Managing Pain in Persons Living with Dementia *Sub‐Module 1* Describe the consequences associated with untreated pain in persons living with dementia Assess for pain, and educate care partners and other caregivers on recognizing signs of pain in persons living with dementia *Sub‐Module 2* Describe nonpharmacological interventions for managing pain in persons living with dementia. Identify when to use pharmacological treatments for treating pain in persons living with dementia	
	**Module 3: Assessing and Managing Behavioral and Psychological Symptoms of Dementia** Behavioral and Psychological Symptoms of Dementia Assessment Apathy Depression Agitation and Aggression Delusions and Hallucinations Sleep Symptoms Motor Behaviors Disinhibition Eating and Feeding *Sub‐Module 1* Describe the consequences associated with untreated behavioral and psychological symptoms of dementia, Describe how to assess for Behavioral and psychological symptoms of dementia Identify approaches to treatment if a symptom is present *Sub‐Modules 2–8* Describe the presentation, assessment, and management of apathy, depression, agitation and aggression, delusions and hallucinations, sleep, motor behaviors, disinhibition, and disinhibition in persons living with dementia. *Sub‐Module 9* Describe challenges and solutions to addressing eating and feeding concerns in persons living with dementia.	
	**Module 4: Delirium Superimposed on Dementia** Describe the significance and presentation of delirium superimposed on dementia Apply methods for assessing potential underlying causes of delirium superimposed on dementia and supporting and educating caregivers about delirium	
	**Module 5: Care Transitions** Foundations of Transitional Care for Persons Living with Dementia Implementing Transitional Care Plans for Persons Living with Dementia *Sub‐Module 1* Define special considerations in caring for persons with dementia and their care partner in the emergency department that support a coordinated, high quality transition to the community. Describe the role and relationship of a care partner to a person living with dementia during care transitions. Identify strategies to prevent emergency department revisits for persons living with dementia. *Sub‐Module 2* Explain the complexities of care transitions from the Emergency Department back to the community Communicate changes and coordinate resources during a care transition with a member of the care team Develop a transitional care plan for persons living with dementia	
	**Module 6: Communicating in the Context of Dementia** Communication with and Support of Persons Living with Dementia Communicating with other members of the interprofessional care team *Sub‐Module 1* Describe the significance of personhood and how it informs communication strategies in persons living with dementia Apply methods for effectively communicating and supporting caregivers of persons living with dementia *Sub‐Module 2* Describe and apply the SBAR technique for communicating with other members of the interprofessional care team.	
Motivational interviewing training	Motivational interviewing techniques to utilize with PLWD and care givers	2 h
Kick off meeting	Post training meeting to ensure implementation and operational readiness	1 h

The modules are broken into short micro‐modules for ease of use. They are interactive and case‐based, include questions throughout, and are accredited for six nursing professional development hours. This training addresses social determinants of health and integrates dementia‐specific assessment instruments, care plans, and care partner education materials. Nurses are trained in performing structured advance care planning discussions. Separately, site leads complete 2 half‐hour modules that provide an overview of the program, its flow, nonpharmacologic and pharmacologic treatments, deprescribing, and expectations of the telephonic lead. A site‐specific resource guide is provided that covers resources in the catchment area of the local health system.

Following completion of the initial training before implementation, nurses and leads are invited to participate together in simulated calls to learn the program's flow, reinforce the language to use when conversing with the dyad, and understand how to handle various easy and complex situations that are likely to present themselves during the program. Each nurse completes a simulated call and workflow before implementing the program and guides each site lead on how to review and provide feedback on a case.

### 5.3 Initial Identification for Telephonic Intervention

Each ED will have EHR‐based analytics that will flag every ED patient who has one prior AD/ADRD ICD‐10 diagnosis code assigned to them within the system. The following alerts will appear in the EHR for the treating team at different points during the ED visit: (1) “This is a PLWD” and (2a) “Please use emergency care redesign procedures,” (2b) “The Community Paramedic team has been notified,” and/or (2c) “The Nurse Telephonic Program has been notified.” To increase reach, ED providers will be able to directly refer individuals who have triggered the clinical decision support alert but have no prior ICD‐10 code to telephonic care. Alternatively, providers can decline the alert by stating that the individual does not have AD/ADRD. As part of their ED discharge instructions and education, a handout as part pf the after‐visit summary will be provided with the nurses' telephone numbers, as well as when to expect an initial call. The ED will confirm the appropriate contact information for the PLWD and their care partner and document it for the telephonic nurse. Dyads will proceed through the telephonic admission process once for the study and followed for 6‐months.

### 5.4 Triadic Initial Encounter Between the PLWD, Care Partner, and Nurse

The initial telephonic encounter will occur within the first 3–5 days of ED discharge. It is expected to last about 60 min and can be divided into smaller segments to be flexible to dyad needs. The nurse first introduces themself and the program, develops a rapport based on the dementia training, and begins the assessment (Table 2). The nurse initially focuses on any immediate needs or significant concerns of the dyad including care transition safety threats, which are identified and using the Hospital‐to‐Home‐Health Transitions Quality (H3TQ) Index [33]. An initial action plan is guided by key principles of optimal transitional care including ensuring follow‐up appointments and coaching on medications, chronic disease management, and cause of ED visits (Table 3) [33, 34, 35, 36, 37]. These parts are prioritized as they are the most likely to lead to building rapport and addressing the greatest initial needs as prioritized by the dyad. Additional initial visit clinical assessments are performed, including functional status, home safety, BPSD, social drivers of health along with understanding care partner–identified positive aspects of care, such as meaningfulness of care partnering and gaining a better understanding of the caregiving team. The nurse discusses which one or two issues they would like to prioritize first outside of imminent care transition safety threats. Once the key issues are determined, the nurse uses the antecedent‐behavior‐consequence‐discussion (ABCD) approach to understand each problem and problem‐solve potential solutions. Evidence‐based care plans that reinforce the training provided in the program are then activated. Dyads are also referred to internal health system and social support resources (e.g., area agency on aging, home healthcare) where needed. Nurses are provided a site‐specific resource manual.

**TABLE 2:** PLWD + care partner assessments, purpose, and timing.

Assessments to be completed across all interventions			NLTC only		
Tool	Topic	Time	Initial call	Second call	Follow‐ups
SDOH*	Social determinants of health	~5 min	Visit 1	No	No
H3TQ	Care transition safety threats	~15 min	Visit 1 ONLY IF NO CPTI Must trigger Care transition Careplan	No	No
QDRS*	Dementia staging	~7 min	Visit 1 Only	No	No
NPI‐Q*	PLWD behavioral symptoms	~5 min	Every visit	Every visit	Every visit
MCSI*	Care partner strain	~3 min	Visit 1	No	Month 3 and last call
Katz ADL*	Functioning	~5 min	Visit 1	No	Month 3 and last call
Katz IADL*	Functioning	~10 min	Visit 1	No	Month 3 and last call
FAM‐CAM*	Delirium	~5 min	Trigger if needed only	Trigger if needed only	Trigger if needed only
Total time			24 min (No H3TQ)	16 min (Warm CPTI Handoff)	8 min (Regular calls)
			30 min (No CPTI)	11 min (Early CPTI Handoff)	23 min (Quarterly)

Abbreviations: FAM‐CAM, Family Confusion Assessment Method; H3TQ, Hospital‐to‐home Transition Quality Index; Katz ADL, Katz Index of Activities of Daily Living; Katz IADL, Katz Index of Independence in Activities of Daily Living; MCSI, Modified Caregiver Strain Index; NPI‐Q, Neuropsychiatric Inventory Questionnaire; QDRS, Quick Dementia Rating Scale; SDOH, Social Determinants of Health.

* Tools common to all three interventions.

**TABLE 3:** Care transitions safety threats.

Information management
Medication reconciliation
Ability to reach healthcare team
Confidence in executing care plans
Timeliness of follow‐up visits
Safety of home environment
Clear expectations of healthcare providers
Ability to manage medications
Understand concerning signs and symptoms

**FIGURE 1:**
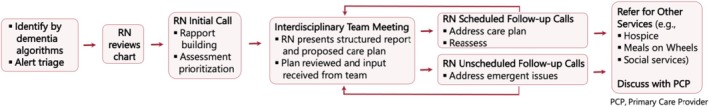
Flow of care.

PLWD who can participate will be invited at the beginning of the first call to share their experiences, feelings, and concerns during the ABCD approach, along with any ideas they have for addressing the concerns. At the end of this first meeting, a follow‐up is scheduled after the next interdisciplinary team meeting, where the plan can be discussed and confirmed (Figure 1). If the patient or care partner has limited English proficiency and the nurse does not speak the care partner's language, the health system's translation service will be used. Although this lengthens the visit duration, in our other work, it has not prevented nurses from completing the initial assessment or developing rapport with the individual or their care partner during initial optimization.

The nurse will complete an initial care plan after the first call. Over the 6 months, nurses must develop and implement at least three standardized problem–intervention–goal care plans in collaboration with the dyad. Advance care planning is required unless already clarified and documented, although this will be readdressed during changes in clinical status. If community paramedicine is not performed, a transitional care plan is required. The other care plan will usually address a BPSD, as identified by the care partner, unless there is a more pressing, agreed‐upon priority. This initial visit is partially modified to prevent overlap where the patient has received care from other programs within ED‐LEAD (emergency care redesign and/or community paramedicine care transitions intervention) prior to an initial call.

### 5.5 Follow‐Up Encounters

Follow‐up encounters will occur at least monthly through the 6‐month follow‐up period, although frequency can increase based on dyad need and nurse capacity. In each follow‐up encounter, the nurse first asks about any changes from the last visit. The nurse then sets the agenda with the dyad for that encounter by (1) completing any required assessments; (2) addressing any plans or interventions discussed at the previous meeting and confirmed in the interdisciplinary team meetings and guiding the care partner through how to perform each intervention; and (3) beginning a discussion about advance care planning if not previously performed or if there is a change in clinical status. If there are significant changes, nurses can always address emergent concerns (e.g., perform a delirium screen) directly if it is within their own scope of practice or with the patient's provider.

### 5.6 Communication With the PLWD'S Designated Provider

When the PLWD is onboarded into the program, the nurse will send a communication (e.g., via EHR, email) to the PLWD's designated provider to inform them that their patient will be participating in the program. The nurse will provide their contact information should the designated provider have any questions. This individual will usually be the PLWD's primary care provider, although this designation will be the dyad's decision. Future communications (e.g., regarding pharmacologic intervention, ordering durable medical equipment, referral for service, follow‐up for unrelated health needs, emergent issues) will then be sent by the nurse to the provider using a modality reflecting the urgency of the issue. Urgent issues where the designated provider cannot be reached will be addressed on a site‐specific basis by the site lead if they are a credentialed provider or by a credentialed designee.

### 5.7 Interdisciplinary Team Meetings

Two types of interdisciplinary team meetings will be held: internal and learning network meetings. Led by the site lead, internal weekly meetings at each site will serve as case conferences, allowing individual cases and feedback on the maintenance of fidelity to the clinical intervention/operations to be reviewed. Patients will be discussed after the first call is completed and then reviewed at regular intervals throughout the period.

Learning network meetings will be 1‐h structured weekly meetings with all active sites. This allows nurses across sites to learn from each other, develop a network of clinicians who are performing the same type of care, and monitor fidelity in a pragmatic way. Learning network meetings are led by a central team of interdisciplinary experts in AD/ADRD care, care transitions, and emergency care. When a new site begins, at least two patients per nurse from that site will be discussed for the first 2 weeks. Thereafter, challenging cases from across sites will be selected for discussion. All cases in the learning network will be deidentified to maintain anonymity. Each case will be presented using a structured format to ensure that pertinent, but not excessive, details are provided and to avoid monopolizing time on a single case. Each discussion will begin with defining age, AD/ADRD stage, care partner relationship to PLWD, any relevant social drivers of health, care transition safety threats, primary BPSD, and plan for treatment using the appropriate care plan, advance care plan status, and goals.

Once a month, in addition to training via case presentations of their own and other nurses in learning network meetings, 1 h will be set aside for development on a specific topic.

These topics are not set in advance, as they are meant to be used to identify new issues brought up during earlier team meetings, for example, assessing suicidality, working with the undomiciled, addressing emergent medical issues, and assessing spiritual needs. These talks will be given by the principal project leads, one of the site leads or nurses if they have specific expertise, or an outside speaker from within participating healthcare systems. Each week, nurses and site PIs will be sent a discipline‐specific email with news about the program, updates, a tool or tip of the week, and a mobile push nudge that includes comparable information in a shorter text format. These email and mobile nudges help to reinforce program elements, and open rates will be tracked.

### 5.8 Setting

Our intervention is triggered pragmatically upon an ED visit. The ED is a place of last resort for PLWD and their care partners and is a sign of unmet needs. It is also a significant predictor of 1‐year mortality in the PLWD [38]. Thus, by targeting dyads who visit the ED, we address a high‐need group with substantial illness burden who are more likely to benefit from such an intervention compared with those who do not use the ED. This nurse‐led telephonic care intervention and its combination with two other interventions will be embedded in 15 health systems representing 79 EDs across the United States. By fully embedding this nurse‐led telephonic care intervention within each health system, while also providing technical assistance for implementation and troubleshooting through an interdisciplinary, multi‐system learning network, we create a system that is more likely to be used in real‐world practice. This increases the generalizability and potential for sustainability.

### 5.9 Nurse Qualifications

The nurses hired for this program will be registered nurses employed by the site and licensed within the state or states in which they will be telephonically working with dyads. They will undergo standard nursing onboarding from their institution.

## 6 Intervention Monitoring

### 6.1 Description of Process Measures

Throughout site implementation, intervention feasibility will be monitored by the number and percent of telephonic care nurses who participate and complete all assigned trainings, the percentage of eligible dyads who complete an initial call with a nurse (70% threshold goal for all sites), and the number and percent of successful handoffs between intervention modalities. Treatment fidelity will be monitored by the number and percent of eligible triadic encounters that adhere to the core components through EHR review by site leads.

## 7 Conclusion

We have described the conceptual basis for a nurse‐led telephonic care intervention. We have outlined the details of its design, including the training, setting, and process measures monitored to ensure delivery and provide a model for other health systems, provider groups, and agencies interested in implementing it. Within the telephonic program of this multi‐factorial pragmatic trial, several highly novel elements will help address substantial knowledge gaps and present methodological innovation that can benefit both this and future studies. Collecting extensive care partner–reported outcomes and social determinants of health will allow us to (1) clinically address risks created by the presence of social drivers of health; (2) examine within‐intervention differences in implementation and changes in care partner–reported outcomes; and (3) explore the impact of PLWD, provider, and site‐level characteristics, including race/ethnicity and neighborhood disadvantage, on quality of implementation.

## Author Contributions

Concept and Design: A.A.B., C.R.G., J.C., M.N.S., K.G., V.T.C., C.J.G., A.I.A. Preparation of Manuscript: All. Acquisition of Data and Interpretation: A.C., A.H., C.R.G., K.G., A.A.B.

## Funding

Research reported in this publication was supported by the National Institute on Aging of the NIH under award number U19 AG078105‐01A1. This research was also funded in part through the NIH/NCI Cancer Center Support Grant P30 CA008748.

## Disclosure

This manuscript is the result of funding in whole or in part by the National Institutes of Health (NIH). It is subject to the NIH Public Access Policy. Through acceptance of this federal funding, NIH has been given a right to make this manuscript publicly available in PubMed Central upon the Official Date of Publication, as defined by NIH. Sponsors of this work had no role in the design, methods, subject recruitment, data collection, analysis, or preparation of this paper.

## Conflicts of Interest

The authors declare no conflicts of interest.

## Linked Article

This publication is part of the special collection titled *Emergency Departments LEading the transformation of Alzheimer’s and Dementia Care (ED‐LEAD)*. To view all articles under this special collection visit https://agsjournals.onlinelibrary.wiley.com/hub/journal/15325415/special‐collections.
